# An extrinsic enzyme-activatable fluorescent probe for high-contrast tumor imaging

**DOI:** 10.1039/d6sc00484a

**Published:** 2026-04-30

**Authors:** Yifang Guo, Yuwei Du, Liru Heng, Huimin Miao, Chen Chen, Qiang Zhang, Jiayi Wang, Yige Shan, Zhang Chen, Li Li

**Affiliations:** a School of Biomedical Engineering (Suzhou), Division of Life Sciences and Medicine, University of Science and Technology of China Hefei 230026 China; b Department of Biomaterials and Stem Cells, Suzhou Institute of Biomedical Engineering and Technology, Chinese Academy of Science (CAS) Suzhou 215163 China chenz@sibet.ac.cn lil@sibet.ac.cn; c Jinan Guoke Medical Technology Development Co., Ltd Jinan 250102 China; d School of Pharmacy, Xuzhou Medical University Xuzhou 221004 China

## Abstract

Malignant tumors pose a major threat to human health, and the effectiveness of treatment largely depends on accurate early diagnosis and complete resection. Although fluorescence imaging offers high sensitivity and enables real-time visualization for intraoperative navigation, conventional fluorescent dyes are limited by photobleaching, poor targeting, and low signal-to-noise (S/N) ratios. These limitations result in false-negative or false-positive outcomes, thereby compromising the precision of surgery and the validity of subsequent treatment decisions. Herein, we developed a fluorescent probe, TMN-CPG, which is specifically activated by carboxypeptidase G2 (CPG2), an enzyme notable for prodrug activation in antibody-directed enzyme prodrug therapy (ADEPT). Upon CPG2-mediated hydrolysis of the TMN-CPG amide bond, the highly fluorescent TMN-NH_2_ is released and subsequently internalized by cancer cells, resulting in tumor-specific fluorescence. In a 4T1 tumor-bearing mouse model, this extrinsic enzyme-activated fluorescence system produced high-contrast tumor imaging. These results indicate that this external enzyme-activatable probe can minimize nonspecific activation, thereby improving the accuracy of tumor imaging.

## Abstract 2

Malignant tumors pose a major threat to human health, and the effectiveness of treatment largely depends on accurate early diagnosis and complete resection.

## Graphical abstract

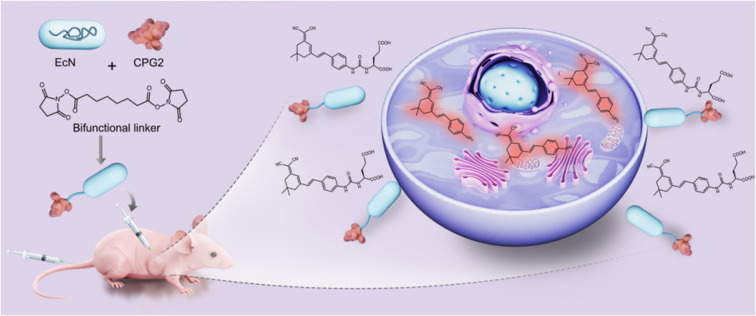

## Introduction

Real-time, high-specificity imaging is critical for precise intraoperative pathological diagnosis of malignant tumors. However, existing clinical imaging techniques, such as computed tomography (CT) and magnetic resonance imaging (MRI), as well as conventional histopathological methods, are insufficient for providing real-time tumor diagnosis during surgery.^1–3^ Substantial potential for *in vivo* tumor visualization has been demonstrated by fluorescence imaging in recent years.^4–7^ Nevertheless, “always-on” probes continuously emit fluorescence independent of their interaction with the target tissues, resulting in moderate or low tumor-to-normal (T/N) tissue ratios and consequently diminished imaging contrast.^8–10^ To improve specificity and diagnostic accuracy, activatable probes have been developed. These probes remain in a quenched state under normal conditions and are activated by disease-associated factors in the tumor microenvironment, leading to pronounced fluorescence emission and markedly improved imaging contrast and signal-to-noise (S/N) ratio.^11–15^

Fluorescence imaging based on enzyme-activated probes has improved the S/N ratios to some extent. The majority of reported activatable probes rely on activation by endogenous enzymes, such as nitroreductase (NTR),^16,17^ aminopeptidase N (APN),^18^ leucine arylamidase (LAP),^19^ and various matrix metalloproteinases (MMPs).^20^ These enzymes are commonly overexpressed in numerous malignancies and possess the capability to cleave specific chemical bonds (*e.g.*, ether, amide, or ester bonds) in the probe, triggering fluorescence turn-on.^21–25^ Despite high sensitivity and specificity, the practical application of endogenous enzyme-activated probes is limited by the presence of these enzymes in non-tumor tissues.^26^ Consequently, off-target probe activation is inevitable, potentially leading to unintended adverse effects. Moreover, the endogenous enzyme concentrations within the tumor microenvironment are inherently limited, which potentially leads to diminished activation efficiency and attenuated signal intensity.

Wong *et al.* have previously established the first extrinsic enzyme-activated photosensitizing system, in which a novel bioconjugation strategy was developed for extrinsic β-galactosidase modification and the enzyme was selectively delivered into cancer cells for lock-and-key-type activation of photosensitizers.^27^ To improve the specificity of tumor imaging, the activation of a fluorescent probe by an extrinsic enzyme facilitates tumor real-time visualization while minimizing background fluorescence signal and nonspecific activation.^28^ In this work, an extrinsic enzyme-specific activatable probe (TMN-CPG) was developed, in which the fluorophore TMN-NH_2_ is masked by a glutamate-based quencher *via* an amide bond. TMN-CPG is specifically activated by the extrinsic enzyme carboxypeptidase G2 (CPG2), a zinc-dependent metalloenzyme known for activating prodrugs in antibody-directed enzyme prodrug therapy (ADEPT).^29–31^ To the best of our knowledge, this study marks the first utilization of CPG2 for fluorescent probe activation in tumor imaging.

**Scheme 1:**
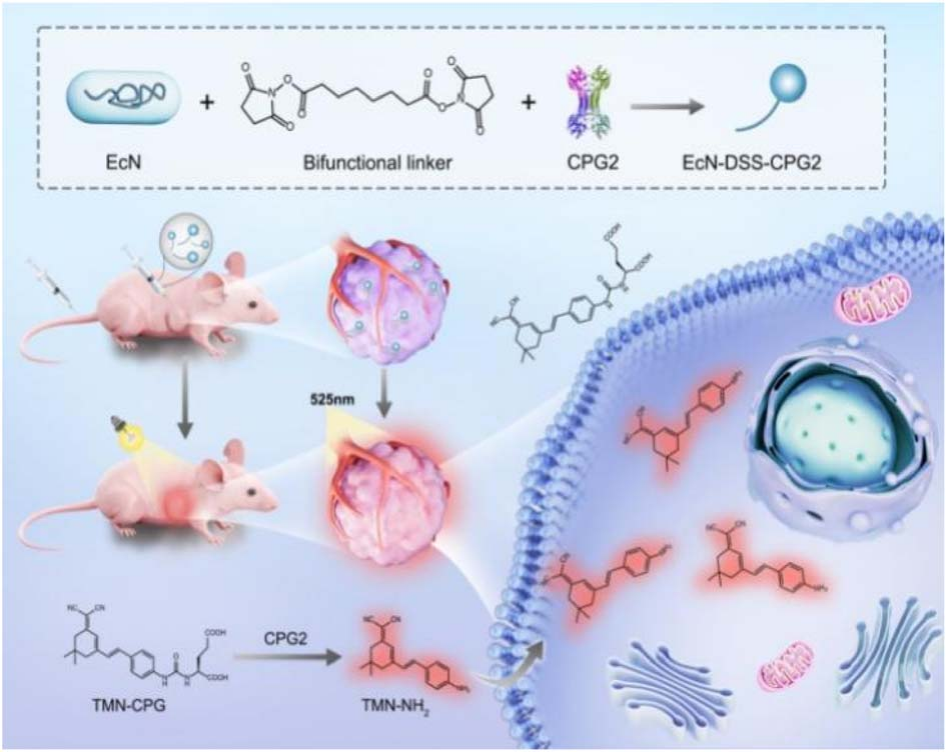
Schematic representation of enhanced FL imaging *in vivo* based on tumor-targeting EcN–DSS–CPG2.

To enhance tumor imaging specificity, a new strategy was developed in which a bacteria-delivered extrinsic enzyme activates a tumor-specific probe for real-time imaging.^32–36^ Studies have shown that certain bacterial vectors can achieve tumor-selective colonization by exploiting the unique microenvironment of solid, tumors, including hypoxia, immune privilege, and nutrient-rich conditions. *Escherichia coli* Nissle 1917 (EcN), a non-pathogenic probiotic, possesses facultative anaerobic metabolism and a well-characterized genetic background, enabling efficient accumulation in hypoxic tumor regions. Its flagellum further enhances penetration into tumor tissue. Meanwhile, the serum-sensitive lipopolysaccharides on the EcN cell membrane facilitate rapid recognition and clearance by the reticuloendothelial system, resulting in short retention time and low background signal in normal organs. In contrast, the nutrient-rich and immunosuppressive tumor microenvironment promotes EcN colonization and proliferation. Based on these advantages, we selected EcN as a biological carrier to supply abundant CPG2 *in situ* for high-contrast imaging.^37–40^ Rather than exclusively targeting surface antigens, our approach takes advantage of bacterial penetration into the tumor core, enabling tumor imaging that surpasses the capabilities of current methods. The intrinsic propensity of bacteria to localize and expand in hypoxic zones of the tumor microenvironment makes this strategy widely applicable for imaging diverse cancers, overcoming the limitations of single-phenotype targeting strategies. For selective tumor delivery of CPG2, EcN was conjugated to CPG2 *via* a disuccinimidyl suberate (DSS) linker to form the EcN–DSS–CPG2 targeting system. After intravenous injection, TMN-CPG reaches the tumor site, where EcN–DSS–CPG2 colonized, releasing the activated fluorophore and resulting in strong fluorescence emission, consequently high-contrast, high S/N real-time tumor fluorescence imaging is enabled (Scheme 1). This approach not only effectively overcomes the inherent lack of specificity associated with endogenous enzyme activation mechanisms but also leverages the capacity of bacterial carriers for selective enrichment and deep tissue penetration within tumor regions, significantly improving imaging resolution and diagnostic reliability.

## Results and discussion

### Probe synthesis and characterization

**Scheme 2:**
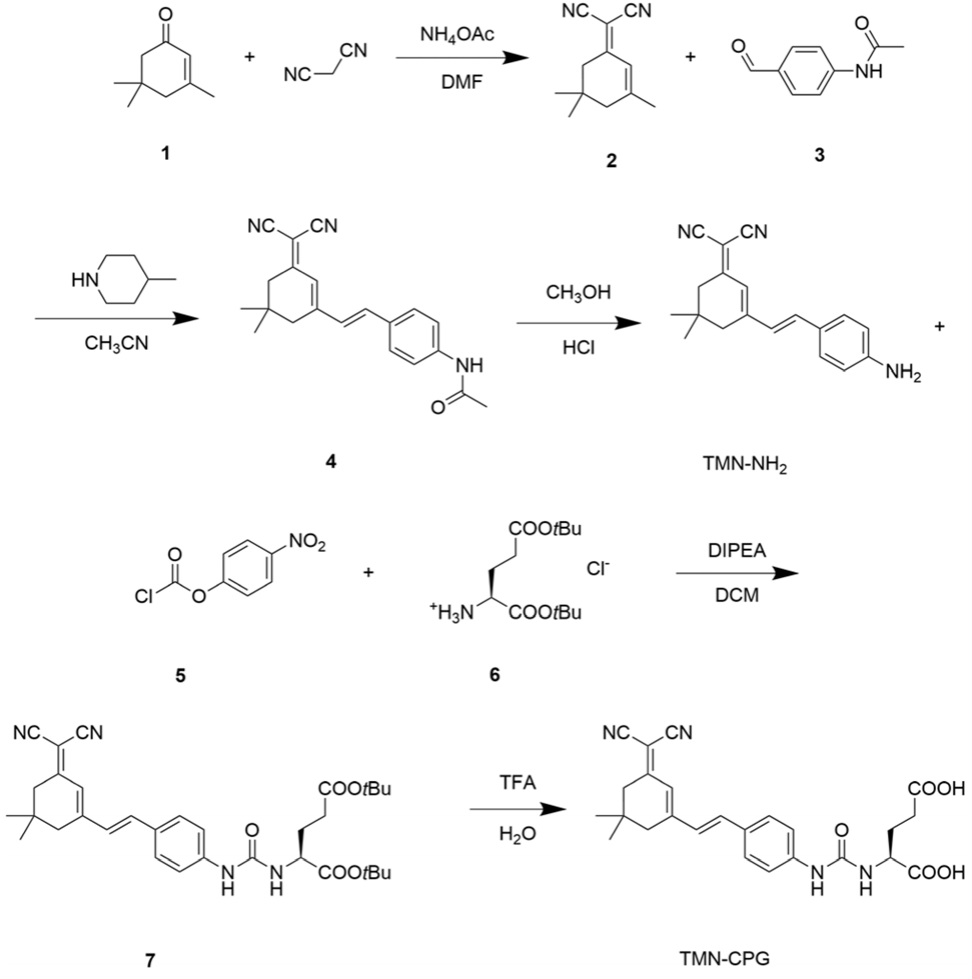
Synthesis route of TMN-CPG.

To achieve *in vivo* visualization of tumors *via* fluorescence imaging, an amide-coupled compound probe, TMN-CPG, was designed as a CPG2-responsive fluorogenic substrate. The enzyme-activatable fluorescent probe TMN-CPG was synthesized *via* a five-step route (Scheme 2). First, malononitrile and isophorone underwent a Knoevenagel condensation to form an extended π-conjugated enone system (compound 2). Michael addition catalyzed by 4-methylpiperidine afforded compound 4. Acidic hydrolysis of compound 4 cleaved the amide bond, generating the primary amine-containing fluorophore TMN-NH_2_. The primary amine of TMN-NH_2_ was then amidated to form an amide bond, affording compound 7. Finally, the *tert*-butyl ester protecting group was removed under strong acid conditions to give the target product TMN-CPG. The structures of TMN-NH_2_ and TMN-CPG were fully characterized by NMR (^1^H, ^13^C) and high-resolution mass spectrometry (HRMS) (Fig. S1–S4).

TMN-NH_2_ is a suitable fluorophore owing to its rigid conjugated framework. The alternating single and double bonds form a large delocalized π-system, providing the orbital space required for electronic transitions. The key amino group in TMN-NH_2_ is an electron-donating group whose lone pair participates in conjugation, enhancing fluorescence. These structural features allow TMN-NH_2_ to efficiently emit photons *via* radiative transition upon photoexcitation, resulting in strong fluorescence. Fluorescence quenching in TMN-CPG is primarily achieved through an amine-based photoinduced electron transfer (PET) mechanism. Once the amino group of TMN-NH_2_ is acylated to form an amide bond, its lone pair is no longer conjugated with the fluorophore but remains localized on the nitrogen atom, acting as an electron donor that quenches the excited-state fluorophore *via* PET. Additionally, the amide bond linking the fluorophore and glutamate is a single bond capable of rotation. The bulky glutamate side chain introduces significant steric hindrance, causing rapid rotation and twisting of the amide bond in the excited state. This disrupts the conjugation between the glutamate moiety and the large π-system, breaking the rigid planar structure. The excited-state energy is dissipated as thermal motion rather than photon emission, leading to fluorescence quenching. The combination of these two mechanisms ensures that TMN-CPG exhibits extremely low background fluorescence and high S/N, making it an excellent protease-activatable probe. When CPG2 cleaves the amide bond in TMN-CPG, the quenching group (glutamate) is detached, removing the electron donor (amide nitrogen) and restoring the rigid planar structure, thereby recovering fluorescence. This activation strategy can be exploited to develop an *in vivo* imaging system for tumor tissues.

**Fig. 1:**
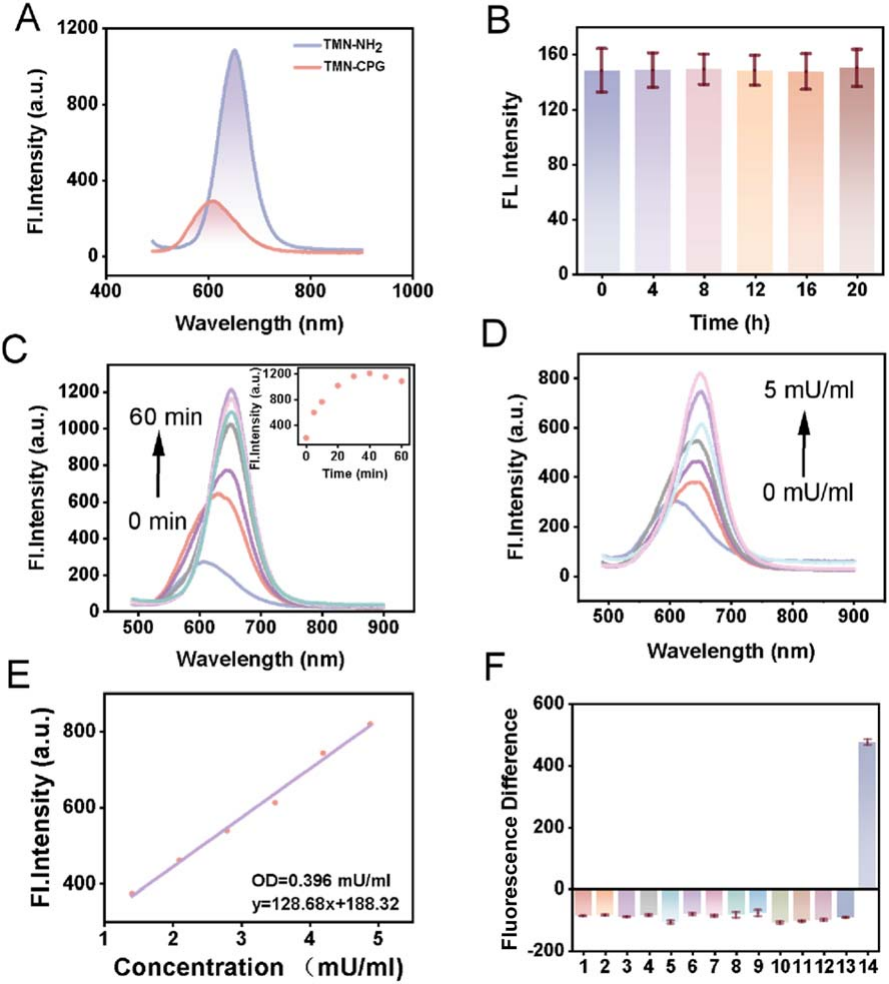
(A) Fluorescence spectra of TMN-NH_2_ and TMN-CPG in PBS buffer (10 µM, pH = 7.3, *λ*_ex_ = 470 nm). (B) Fluorescence intensity of TMN-CPG (10 µM) was recorded in PBS solution as a function of time. Error bars are for *n* = 3. (C) Fluorescence (*λ*_ex_ = 470 nm) spectra of TMN-CPG (10 µM) in the presence of CPG2 (5 mU mL^−1^) in PBS (pH 7.3) at 37 °C over a period of 60 min. (D) Fluorescence spectrometric titration experiments. (E) The linear functional relationship between the fluorescence intensity of 650 nm and low concentration CPG2. (F) Selectivity of TMN-CPG towards different analytes in PBS buffer. Except for special statement, all other concentrations were 1 mM. Insert (1) blank, (2) Ca^2+^, (3) Mg^2+^, (4) Zn^2+^, (5) glutathione, (6) H_2_O_2_, (7) cysteine, (8) vitamin C, (9) dithiothreitol, (10) Cl^−^, (11) HCO_3_^−^, (12) SO_4_^2−^, (13) biotin, (14) CPG2 (5 mU mL^−1^). Error bars are for *n* = 3.

To verify that TMN-CPG exhibits an initial “fluorescence-off” state, fluorescence spectra of TMN-CPG and TMN-NH_2_ were studied in PBS. As shown in Fig. 1A, under 470 nm excitation, the maximum emission wavelengths were 610 nm for TMN-CPG and 650 nm for TMN-NH_2_. As expected, the fluorescence signal of TMN-CPG at 650 nm was negligible compared to that of TMN-NH_2_, indicating that incorporation of the glutamate group effectively eliminates background signal, suggesting that TMN-CPG can serve as a near-infrared probe for deep-tissue *in vivo* imaging.

The photostability of a fluorescent probe is one of the key factors determining its suitability for long-duration imaging applications. To quantitatively evaluate the photobleaching resistance of TMN-NH_2_, the probe (10 µM in PBS) was continuously exposed to a 470 nm light source (60 mW cm^−2^), and its fluorescence intensity at 650 nm was recorded every 4 h for a total duration of 20 h. As shown in Fig. S5, TMN-NH_2_ exhibited minimal photobleaching, retaining more than 90% of its initial fluorescence intensity after 20 h of continuous irradiation. The high photostability of TMN-NH_2_ provides a crucial guarantee for its application in clinical settings requiring prolonged and continuous illumination, such as fluorescence-guided surgery, positioning it as an ideal candidate probe for precise tumor imaging.

The optical response of TMN-CPG to CPG2 was investigated by monitoring changes in fluorescence spectra. CPG2 (5 mU mL^−1^) was pre-incubated in PBS (pH 7.3) at 37 °C for 10 min to activate it, followed by addition of probe TMN-CPG (10 µM) and ZnCl_2_ (200 µM). Fluorescence spectra were recorded at different incubation times. In the absence of CPG2, TMN-CPG remained non-fluorescent over time, indicating probe stability in the buffer (Fig. 1B). In contrast, with 5 mU per mL CPG2, the emission wavelength of TMN-CPG gradually shifted from 610 nm to 650 nm and inducing a time-dependent increase in emission at 650 nm, reaching a plateau at 30 min (Fig. 1C). An enzyme concentration titration experiment was performed to evaluate the quantitative detection capability of TMN-CPG for CPG2. A concentration series of CPG2 (0–5 mU mL^−1^) was incubated with TMN-CPG (10 µM, 37 °C, 30 min). As shown in Fig. 1D, the fluorescence intensity of TMN-CPG increased with CPG2 concentration. A good linear relationship was observed between the fluorescence intensity at 650 nm and CPG2 concentration in Fig. 1E, and the linear regression equation for CPG2 detection is *y* = 128.68*x* + 188.32 (*R*^2^ = 0.993), where *y* represents the fluorescence intensity at 650 nm and *x* represents the CPG2 concentration (mU mL^−1^). The limit of detection (LOD) was calculated to be 0.396 mU mL^−1^ using 3*σ*/slope (where *σ* was the standard deviation of blank measurements). These results indicated that CPG2 can activate the fluorescence of TMN-CPG with high sensitivity.

High specificity is crucial for practical application in complex physiological environments, especially in bioimaging and disease diagnosis. Accordingly, the fluorescence response of TMN-CPG to various potential interferents (including enzymes, biomolecules, inorganic salts) was studied in PBS. Upon activation of TMN-CPG, the emission wavelength shifts from 610 nm to 650 nm. Therefore, the fluorescence enhancement of the probe following enzyme action was assessed by subtracting the fluorescence intensity at 610 nm from that at 650 nm. As shown in Fig. 1F, no fluorescence enhancement was observed with MgCl_2_, CaCl_2_, ZnCl_2_, cysteine (Cys), dithiothreitol (DTT), glutathione (GSH), vitamin C, H_2_O_2,_ NaCl, NaHCO_3_, Na_2_SO_4_ or biotin. Only CPG2 treatment had a significant enhancement of fluorescence. These results demonstrate that CPG2 specifically activates TMN-CPG with high selectivity.

### Theoretical simulations of TMN-NH_2_ and TMN-CPG using TDDFT method

**Fig. 2:**
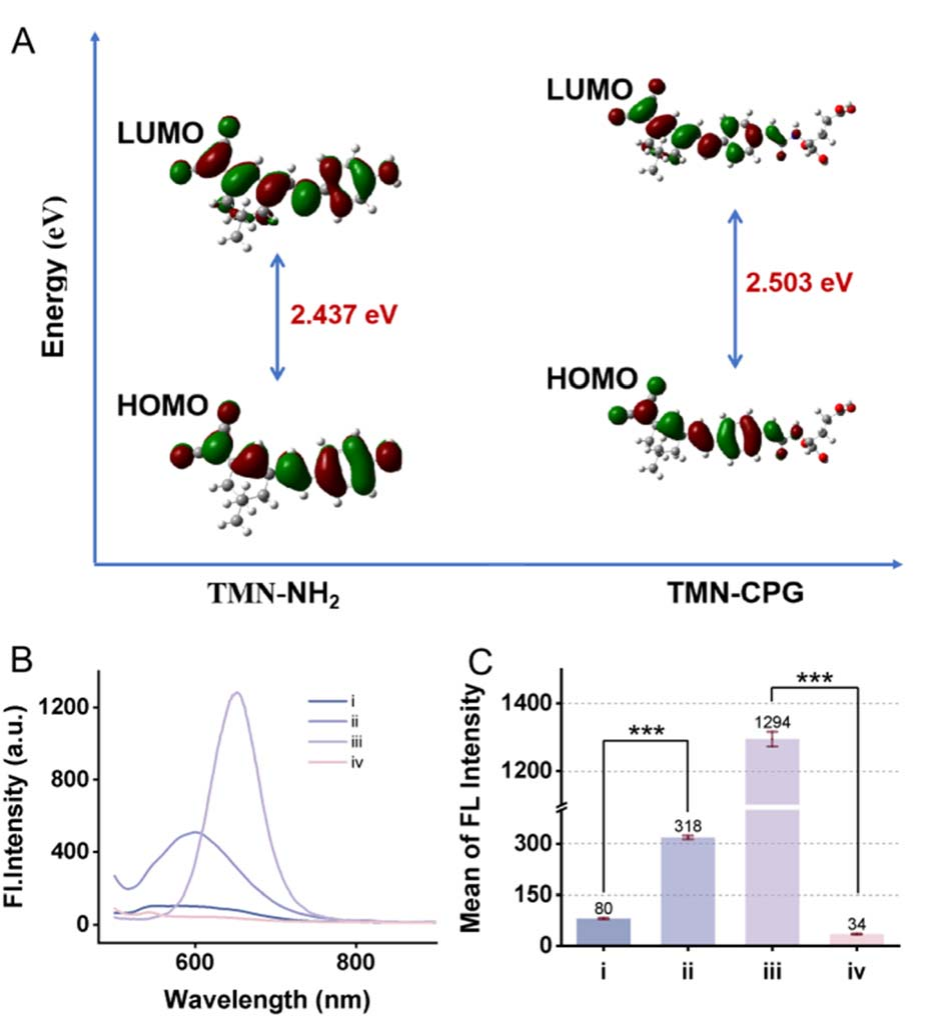
(A) Orbital energies of TMN-NH_2_ and TMN-CPG. (B) Fluorescence spectra (*λ*_ex_ = 470 nm) of the octanol phase and PBS phase of ((i) and (ii)) TMN-CPG and ((iii) and (iv)) TMN-NH_2_. (C) A histogram of the FL intensity of (i)–(iv) at an emission wavelength of 650 nm. Error bars are for *n* = 3.

We performed theoretical simulations on two molecules, TMN-NH_2_ and TMN-CPG, employing the time-dependent density functional theory (TDDFT) method. The B3LYP functional was utilized, and the 6-311++G(d,p) basis set was applied for all calculations. Initially, the molecular structures of both compounds were optimized in their first excited state (S_1_) using the TDDFT approach, and the corresponding orbital energies were computed. Subsequently, the vertical transition energies were determined, which corresponds to the emission energies of the molecules. As shown in Fig. 2A, the calculated results show that the energy gap (the difference between the highest occupied molecular orbital (HOMO) and the lowest unoccupied molecular orbital (LUMO)) for TMN-NH_2_ is 2.437 eV, while for TMN-CPG, it is 2.503 eV. The emission energy of TMN-NH_2_ was calculated to be 598.8 nm, whereas for TMN-CPG, it is 570.4 nm. These computational results are consistent with experimental trends, suggesting that following interaction with the enzyme, the fluorescence of TMN-CPG undergoes a redshift.

### Response kinetics and cellular imaging

An ideal tumor imaging probe should not only produce signals specifically at the tumor site but also have its *in vivo* distribution precisely regulated to prevent nonspecific accumulation in non-target areas. Therefore, the lipophilicity of TMN-CPG and TMN-NH_2_ was determined using the octanol/PBS shake-flask method to predict their behavior in biological systems. As shown in Fig. 2B and C, the log *P* value of TMN-CPG in the octanol phase was −0.6, while that of TMN-NH_2_ significantly increased to 1.58. The lipophilicity measurements confirmed that the probe in its non-activated state exhibits ideal hydrophilic properties, while the activated probe can efficiently enter and be retained within cells, achieving spatiotemporal synchronization of signal generation and retention. This “cell-anchoring” strategy effectively addresses the issue of signal loss that may occur with extracellularly activated probes due to bodily fluid washout or rapid metabolism, ensuring stable and persistent imaging signals. It offers a robust solution to the key challenges in *in vivo* tumor imaging, namely high S/N ratio and high specificity.

**Fig. 3:**
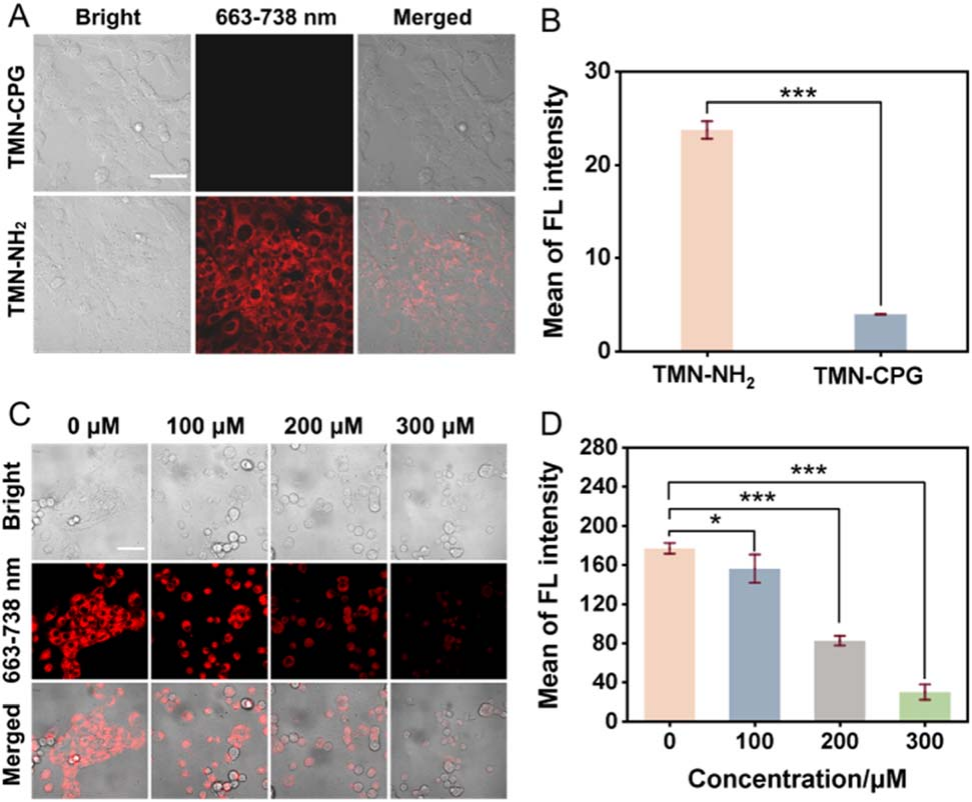
(A) Confocal fluorescence images of 4T1 cells after incubation with TMN-NH_2_ (10 µM) or TMN-CPG (10 µM) for 30 min. Scale bar = 50 µm. (B) Comparison of the intracellular fluorescence intensities in 4T1 cells after incubation with TMN-NH_2_ (10 µM) or TMN-CPG (10 µM) for 30 min. Error bars are for *n* = 4. (C) Confocal fluorescence images of 4T1 cells pre-treated with different concentrations of T10678 and incubated with TMN-CPG (10 µM) and CPG2 (10 mU mL^−1^) for 30 min. Scale bar = 50 µm. (D) Histogram of the quantified FL intensities from 4T1 cells pre-treated with different concentrations of T10678 and incubated with TMN-CPG (10 µM) and CPG2 (10 mU mL^−1^) for 30 min. Error bars are for *n* = 3. Scale bar = 20 µm.

To evaluate the cellular imaging capability, 4T1 cells were incubated with TMN-NH_2_ or TMN-CPG (10 µM, 30 min). As shown in Fig. 3A and B, cells treated with TMN-CPG showed almost no fluorescence, while those treated with TMN-NH_2_ exhibited strong fluorescence, approximately 6 times higher than TMN-CPG-treated cells. These results indicate that the activated TMN-NH_2_ has good cellular permeability and high S/N ratio. The significant difference in fluorescence intensity between TMN-CPG and TMN-NH_2_ at the cellular level demonstrated that this probe activation strategy can markedly enhance the contrast of tumor cell imaging.

4T1 cells were pre-treated with T10678, which is a specific CPG2 inhibitor for 30 min, followed by adding 10 µM TMN-CPG and 10 mU per mL CPG2 for another 30 min. As shown in Fig. 3C, preincubations with T10678 significantly suppressed fluorescence intensity in 4T1 cells compared to the control. The fluorescence intensity of TMN-CPG was reduced by 83.3% with the presence of 300 µM T10678 compared to the control group without T10678, and fluorescence intensity decreased gradually with increasing T10678 concentration (Fig. 3D). The result confirmed that the fluorescence signal was indeed induced by CPG2 catalysis, providing ideal conditions for subsequent *in vivo* bioimaging.

To investigate the specific internalization mechanism by which the activated fluorophore TMN-NH_2_ enters 4T1 cells, a pharmacological inhibition assay was performed to systematically characterize its endocytic pathways. Cells were pre-incubated for 1 h with chlorpromazine (10 µg mL^−1^, an inhibitor that specifically suppresses clathrin-mediated endocytosis), genistein (100 µM, an inhibitor that blocks caveolae-dependent lipid raft-mediated endocytosis), and amiloride (50 µM, an inhibitor that inhibits macropinocytosis), followed by co-incubation with TMN-NH_2_ (10 µM) for an additional 30 min. Cells treated with DMSO served as the negative control. As shown in Fig. S6 and S7, no significant reduction in intracellular fluorescence intensity was observed in cells pretreated with any of the three inhibitors compared to the DMSO control group. These results indicate that clathrin-mediated endocytosis, caveolae-mediated endocytosis, and macropinocytosis are not involved in the cellular internalization of TMN-NH_2_. The TMN-NH_2_ molecule possesses moderate lipophilicity (log *P* = 1.58) and bears a primary amino group; such cationic lipophilic fluorescent probes are known to enter cells *via* non-canonical pathways or direct passive diffusion across the plasma membrane. Based on the above inhibitor results and molecular structure analysis, it is proposed that TMN-NH_2_ enters 4T1 cells primarily through passive diffusion or a non-canonical transmembrane translocation mechanism that is independent of classical endocytosis. This finding demonstrates that, upon enzymatic cleavage and release, TMN-NH_2_ is able to rapidly enter cancer cells and be retained for a prolonged period, thereby achieving signal amplification and sustained imaging at the tumor site.

**Fig. 4:**
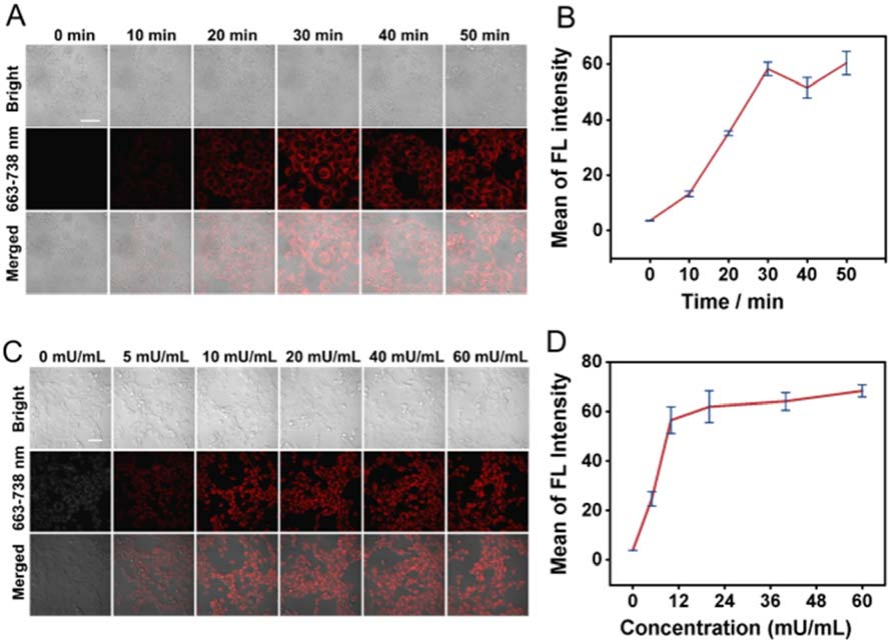
(A) Confocal fluorescence images of 4T1 cells incubated with TMN-CPG (10 µM) and CPG2 (10 mU mL^−1^) and for different incubation times. Scale bar = 50 µm. (B) Histogram of the quantified FL intensities from 4T1 cells incubated with TMN-CPG (10 µM) and CPG2 (10 mU mL^−1^) and for different incubation times. Error bars are for *n* = 3. (C) Confocal fluorescence images of 4T1 cells incubated with different concentrations of CPG2 and TMN-CPG (10 µM) for 30 min. Scale bar = 50 µm. (D) Histogram of the quantified FL intensities from 4T1 cells incubated with different concentrations of CPG2 and TMN-CPG (10 µM) for 30 min. Error bars are for *n* = 5.

The activation of TMN-CPG by CPG2 was further studied at the cellular level using confocal microscopy. 4T1 cells were incubated with TMN-CPG (10 µM) and CPG2 (10 mU mL^−1^) for different times to determine the optimal time for enzymatic reaction. Confocal images Fig. 4A and B showed that fluorescence intensity increased with incubation time, reaching a maximum at 30 min. The enzyme dose-dependent of TMN-CPG activation was then investigated. When incubated for 30 min, cellular fluorescence increased with CPG2 concentration and plateaued at 10 mU mL^−1^ (Fig. 4C and D). Thus, 10 mU per mL CPG2 and 30 min incubation time were selected for subsequent *in vivo* studies.

**Fig. 5:**
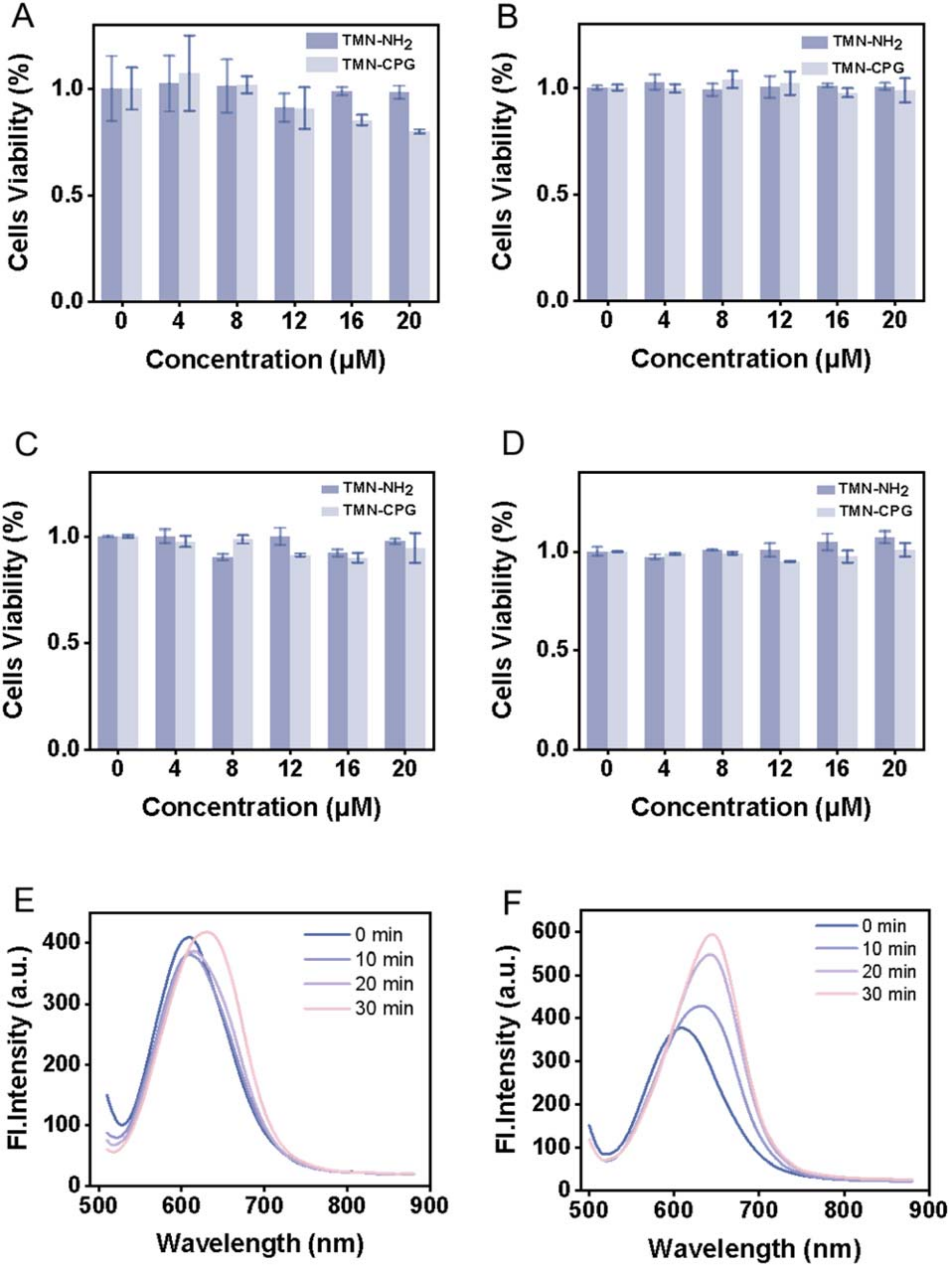
(A–D) WST-1 assays of TMN-CPG in 4T1 cells, 3T3 cells, HeLa cells and L929 cells. Error bars are for *n* = 3. (E) The time response of TMN-CPG (10 µM) in response to EcN in PBS buffer solution. (F) The time response of TMN-CPG (10 µM) in response to EcN–DSS–CPG2 in PBS buffer solution.

Good biocompatibility is a prerequisite for probe application. Accordingly, the biocompatibility of TMN-NH_2_ and TMN-CPG was evaluated, and cytotoxicity was assessed using the WST-1 assay. As shown in Fig. 5A–D, TMN-NH_2_ and TMN-CPG did not significantly reduce the viability of various cells from 0 to 20 µM, indicating that TMN-CPG can serve as a reliable tool for live cell and *in vivo* imaging.

### Construction of EcN–DSS–CPG2 and CPG2 activity assay

To conjugate CPG2 to EcN for a composite delivery system, a homobifunctional crosslinker DSS was selected as the linker. One *N*-hydroxysuccinimide (NHS) ester group of DSS reacts with primary amines (NH_2_) on CPG2 to form a stable amide bond. The surface of EcN is rich in lysine residues, and the other NHS ester of DSS reacts with the ε-amino group of lysine to form the EcN–DSS–CPG2 covalent complex. This approach enables a stable covalent conjugation between EcN and CPG2, suitable for constructing a bacterial–enzyme composite system. Detailed conjugation procedures are provided in the SI. The fluorescence response of TMN-CPG (10 µM) to EcN–DSS–CPG2 or EcN (both at 1 × 10^7^ CFU mL^−1^) was evaluated in PBS (30 min). As shown in Fig. 5E and F, EcN did not activate TMN-CPG, while EcN–DSS–CPG2 produced a time-dependent increase over 30 min. This confirms that EcN–DSS–CPG2 and can specifically activate TMN-CPG. It is anticipated that EcN–DSS–CPG2 will afford superior S/N in tumor tissue, making it suitable for *in vivo* tumor imaging. The results showed that as the incubation time was extended to 30 minutes, the fluorescence intensity of TMN-CPG reached 588. Using the previously established linear relationship between fluorescence intensity at 650 nm and CPG2 concentration (*y* = 128.68*x* + 188.32), the concentration of CPG2 in the reaction system was calculated to be 0.000834 µM. Further combined with the number of bacteria, the average number of CPG2 molecules conjugated per EcN cell was calculated to be approximately 5.02 × 10^4^. This result indicates that the DSS-mediated covalent conjugation strategy can efficiently anchor a large number of CPG2 enzyme molecules onto the EcN surface, providing a sufficient reservoir of active enzymes for subsequent local enrichment at the tumor site.

**Fig. 6:**
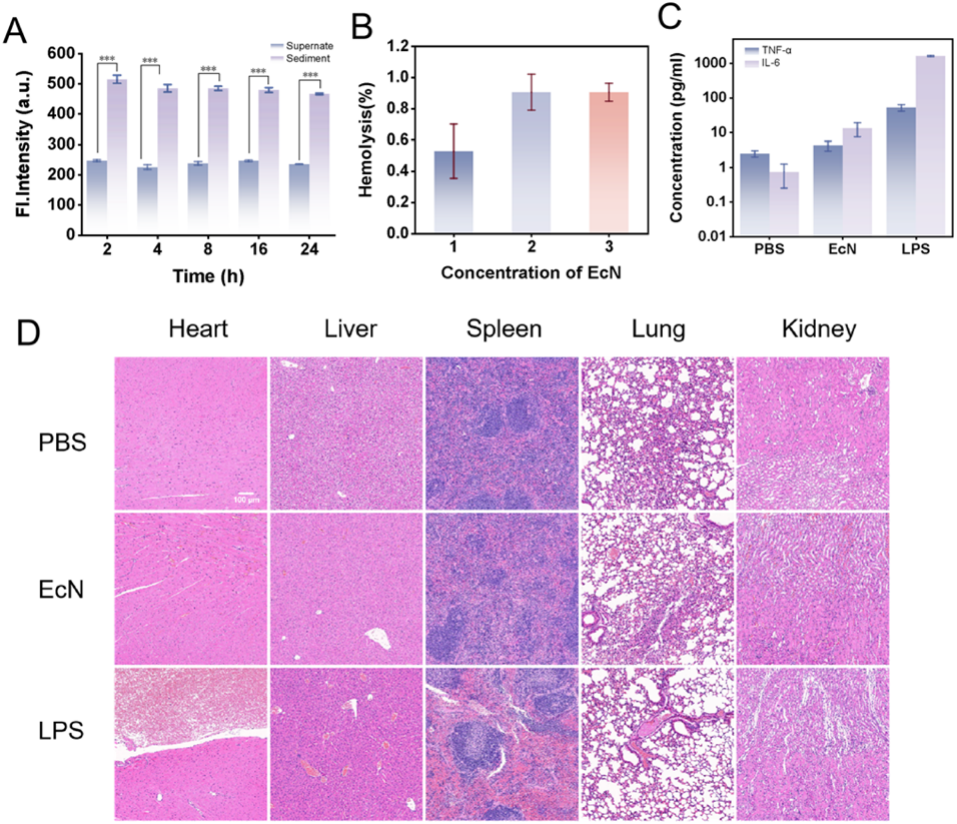
(A) Fluorescence intensity of TMN-CPG (10 µM) incubated with either the supernatant or the resuspended pellet of the EcN–DSS–CPG2 complex at indicated time points was recorded in PBS solution as a function of time. Error bars are for *n* = 3. (B) Hemolysis rate of 4T1 cells co-incubated with various concentrations of EcN. Insert (1) 1 × 10^6^ CFU mL^−1^, (2) 1 × 10^7^ CFU mL^−1^, (3) 1 × 10^8^ CFU mL^−1^. Error bars are for *n* = 3. (C) Changes in immune factors in Balb/c mice under different treatment conditions. Error bars are for *n* = 3. (D) H&E staining of main immune tissue of Balb/c mice.

Subsequently, to evaluate the stability of the covalent linkage of the EcN–DSS–CPG2 complex under physiological conditions, the complex was incubated in PBS buffer and 50% fetal bovine serum (FBS) at 37 °C. At various time points, samples were collected and centrifuged, and CPG2 enzymatic activity was measured in both the supernatant and the resuspended pellet (Fig. 6A). The results showed that even after 2 h of incubation in PBS and 50% FBS, 81.6% of CPG2 activity remained associated with the bacterial pellet; after 24 h of incubation, this proportion was still maintained at 69.7%. These findings confirm that the covalent bond formed *via* the DSS crosslinker exhibits high stability under physiological conditions, effectively resisting hydrolysis by serum proteins and buffer media, thereby ensuring that CPG2 enzymes remain anchored to the EcN surface without significant detachment during circulation and within the tumor microenvironment. This excellent stability is crucial for ensuring the sustained retention of the enzyme at the tumor site and achieving continuous activation of the circulating probe TMN-CPG, laying a solid foundation for the *in vivo* application of bacterial vector-based exogenous enzyme delivery strategies.

### Precise tumor targeting and biosafety of EcN in tumor-bearing mice

Dai *et al.* transferred a luciferase-expressing reporter plasmid into EcN, after which the bacteria were injected into tumor-bearing mice. The distribution of EcN was observed using a small animal imaging system 7 days later, and the results showed that no signal was detected in major immune tissues except for the tumor tissue.^41^ Li *et al.* evaluated the *in vivo* biodistribution of bacteria using an engineered EcN strain expressing a bioluminescent reporter gene, demonstrating that the bacteria colonized exclusively in the tumor, with negligible presence in the heart, liver, spleen, lungs, or kidneys.^42^ These findings collectively confirmed the precise tumor-targeting capability of EcN. It has been shown that EcN is rapidly cleared from the bloodstream of healthy mice within 12 h without causing significant body weight changes. Furthermore, an *in vitro* hemolysis assay confirmed that EcN does not induce significant red blood cell lysis (hemolysis rate < 5%) even at a high dose of 5 × 10^8^ CFU mL^−1^ (Fig. 6B), providing additional evidence for its biosafety. These results indicate that EcN does not elicit long-term cytotoxic effects and possesses a favorable biosafety profile, making it suitable for *in vivo* administration. To further evaluate the biosafety of EcN, healthy mice were injected with 1 × 10^7^ CFU of EcN. Blood samples collected 6 h post-injection were analyzed for inflammatory markers, revealing that TNF-α and IL-6 levels remained within the normal range (Fig. 6C). These results suggested that the bacteria did not trigger systemic inflammation. Histopathological analysis (H&E staining) of excised tissues performed 24 h post-injection revealed no significant differences in the pathophysiology of major immune organs between EcN-treated and PBS-treated mice (Fig. 6D), indicating that EcN did not induce acute toxicity. Collectively, these comprehensive analyses demonstrate that EcN serves as a safe and effective tumor-targeting system, achieving precise tumor localization and a high degree of safety, thereby reinforcing its potential for safe and effective clinical translation in fluorescence-guided cancer surgery.

### *In vivo* experiments

Inspired by the desired activatable emission in responsive to CPG2, the capability of TMN-CPG for visualization of tumor *in vivo* was then investigated. 4T1 cells (2.5 × 10^6^ cells) were transplanted around the axilla of the right limb of female nude Balb/c mice to establish the animal research model. Tumor-bearing mice were randomized into four groups: (1) intravenous (IV) injection of 2 mM TMN-CPG (150 µL in PBS) and intratumoral (IT) injection of native CPG2 (tumor concentration is 100 mU mL^−1^) or PBS. (2) IV injection of native CPG2 (systemic concentration is 100 mU mL^−1^) or PBS and IT injection of TMN-CPG (150 µL). (3) IV injection of TMN-CPG (150 µL) and IT injection of EcN–DSS–CPG2 (2 × 10^7^ CFU) or PBS. (4) IV injection of Cyanine 5 (Cy5) (systemic concentration is 10 µM). Groups 1 and 2 served as controls. Since EcN is serum-sensitive, IV injection would lead to rapid clearance by the reticuloendothelial system (RES) before tumor colonization. Leaky tumor vasculature and impaired lymphatic drainage favor retention, therefore, intratumoral injection was used to ensure sufficient bacterial colonization.

**Fig. 7:**
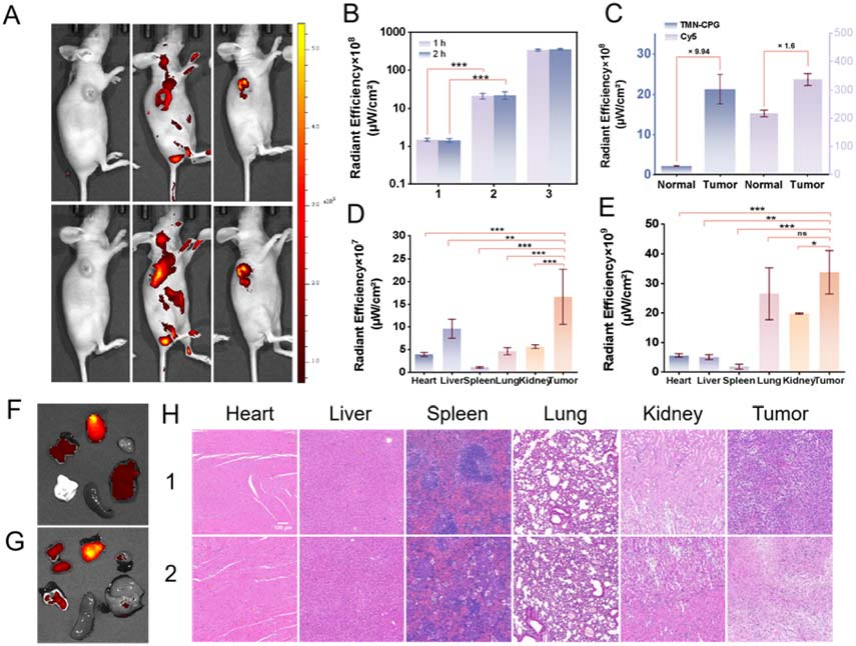
(A) *In vivo* imaging of 4T1 xenograft-tumor-bearing nude Balb/c mice at 1 and 2 h after (1) intravenous (IV) injection of TMN-CPG and intratumoral (IT) injection of PBS; (2) IV injection of Cy5. (3) IV injection of TMN-CPG and IT injection of EcN–DSS–CPG2. *λ*_ex_ = 470 nm and *λ*_detection_ = 650 nm. (B) Corresponding quantitative comparison of the three groups in the tumor regions. Error bars are for *n* = 3. (C) Corresponding quantitative comparison of fluorescence between tumor and normal tissues in two groups of mice. Error bars are for *n* = 3. (D) Normalized fluorescence intensities of different organs from group 3 mice. Error bars are for *n* = 3. (E) Normalized fluorescence intensities of different organs from group 2 mice. Error bars are for *n* = 3. (F) *Ex vivo* images of the resected tumor and organs (including liver, spleen, heart, kidney, and intestine) obtained from group 3 mice. (G) *Ex vivo* images of the resected tumor and organs (including liver, spleen, heart, kidney, and intestine) obtained from group 2 mice. (H) H&E staining of tumor tissue and main immune tissue of tumor-bearing mice. (1) IV injection of TMN-CPG and IT injection of PBS; (2) IV injection of Cy5.

*In vivo* imaging was performed on a small animal imaging system at 670 nm emission at different time points after treatment. As shown in Fig. S8, no fluorescence was detected in PBS-treated groups, indicating that the probe is not activated by the intrinsic tumor microenvironment and that CPG2 is not endogenous. Group 1 showed no fluorescence signal because native CPG2 lacks targeting ability and when it is injected into the tumor, it will quickly leak back into the circulation *via* tumor blood vessels and be diluted and cleared. This result indicates that the enzyme must be anchored in the tumor to maintain effective concentration for continuous activation of circulating probes. In group 2, obvious tumor-site fluorescence confirmed effective *in vivo* cleavage and activation of TMN-CPG by CPG2, supporting the applicability of this probe for *in vivo* application. Group 3 images showed bright tumor illumination (Fig. 7A). Quantitative fluorescence analysis of the *in vivo* imaging data from mice in group 3 revealed that the tumor fluorescence intensity in mice injected with EcN–DSS–CPG2 was significantly higher than that in the PBS control group. This result indicated that the synthesized EcN–DSS–CPG2 was able to selectively colonize the tumor site and retained sufficient enzymatic activity under physiological conditions to activate the fluorescence emission of TMN-CPG at the tumor site. Notably, the fluorescence intensity of the activated probe exhibited almost no attenuation within 2 h (Fig. 7B), ensuring the stability of tumor imaging. By normalizing the tumor fluorescence signal against the surrounding skin background, the S/N ratio was calculated to be as high as 9.94 at 2 h post-injection, which was significantly higher than that of conventional non-activatable dyes (S/N ratio of only 1.6 for Cy5), demonstrating that this system enables high-contrast identification of cancerous tissue (Fig. 7C). These results validated the extrinsic enzyme-activated probe strategy as a high-contrast, real-time tumor-labeling platform within the tumor microenvironment and provide evidence for imaging and targeted-therapy development.

To elucidate the biodistribution and metabolic pathway of the probe, *ex vivo* fluorescence imaging was performed on major organs (heart, liver, spleen, lungs, kidneys) and tumors collected from group 3 mice immediately 2 h after probe injection. As shown in Fig. 7D and F, the strongest fluorescence signal was observed in the tumor tissue, whereas substantial signals were also detected in the liver and kidneys, with only negligible background fluorescence in the other organs. This distribution pattern indicated that the activated fluorophore TMN-NH_2_ is primarily cleared *via* the hepatobiliary and renal dual-clearance pathways. This clearance profile is consistent with the molecular polarity (log *P* = 1.58) and pharmacokinetic properties of TMN-NH_2_. The dual-clearance mechanism not only ensures a high signal-to-noise ratio during the imaging window but also mitigates the potential toxicity risks associated with long-term retention of the fluorophore.

Furthermore, histopathological examination (H&E staining) was performed on normal organs and tumor tissues collected from the same cohort of mice 24 h after probe injection. The results showed mild inflammatory changes within the tumor tissue of EcN-treated mice, characterized by increased infiltration of macrophages and neutrophils. This finding was likely attributable to the local innate immune response elicited by bacterial colonization. In contrast, no significant histopathological abnormalities (necrosis, fibrosis, or acute toxicity) were observed in the major immune organs (heart, liver, spleen, lungs, kidneys) compared with normal mice (Fig. 7H). These results are consistent with the absence of significant elevation in serum pro-inflammatory cytokines (TNF-α, IL-6) and further support the biosafety of the EcN–DSS–CPG2 system. Collectively, the above data demonstrate that our bacteria-mediated extrinsic enzyme-activatable fluorescent probe strategy achieves high-contrast, stable, and safe tumor-specific imaging, exhibiting strong potential for clinical translation.

### System advantages and clinical translation potential

In recent years, extrinsic enzyme-activatable imaging systems have attracted considerable attention due to their ability to effectively circumvent the nonspecific activation inherent to endogenous enzyme-based strategies. Wong *et al.* first reported an extrinsic enzyme-activated photosensitizer system for precise photodynamic therapy, which demonstrated the significant advantages of the extrinsic enzyme activation strategy in reducing nonspecific activation. In a tumor-bearing nude mouse model, a S/N of approximately 4 was achieved, and kinetic analysis revealed that the fluorescence signal reached a plateau at 90 minutes, with an enzyme concentration of 6 U mL^−1^.^27^ This work provided the first proof-of-principle validation of the extrinsic enzyme activation strategy for precision tumor therapy. However, the system relies on chemical linker-mediated enzyme delivery, and its targeting efficiency is constrained by the expression level of surface antigens on tumors; moreover, the enzyme may be inactivated in circulation due to hydrolysis or proteolytic degradation. Meng *et al.* utilized an engineered EcN strain that continuously expressed endogenous NTR as a biomarker to activate an exogenous NTR-responsive fluorescent probe. A 3.15 fold enhancement of fluorescence signal was achieved in bacteria-colonized tumors. Nevertheless, this strategy still relies on the NTR enzyme expressed by the engineered bacteria themselves, and its activation specificity is limited by the expression level of the bacterial enzyme.^28^ Moreover, this system requires genetic engineering of the bacteria to introduce the exogenous NTR gene, introducing a certain level of genetic modification complexity.

In contrast, the EcN-mediated CPG2 delivery system exploits the intrinsic tumor-colonizing ability of bacteria in hypoxic regions to achieve *in situ* enrichment of the exogenous enzyme, avoiding complex chemical synthesis and purification steps. Our strategy is independent of tumor surface antigen expression levels and does not require complex genetic engineering of the bacteria. Instead, a chemical conjugation strategy is employed to directly anchor the exogenous enzyme CPG2 onto the EcN surface, and signal amplification is achieved through an enzymatic cascade. Kinetic analysis of this system demonstrated that the fluorescence signal reached a plateau within 30 minutes, requiring only 5 mU mL^−1^ of enzyme, and an S/N of 9.94 was achieved, with both activation kinetics and imaging contrast being significantly superior to those of the systems described above. More importantly, our strategy employs a completely exogenous enzyme (CPG2) as the activation trigger, whose expression and delivery are entirely independent of bacterial metabolism and the endogenous tumor environment, thereby offering higher controllability and tumor applicability. Collectively, the strategy of EcN-mediated extrinsic enzyme activation exhibits unique advantages in enzyme delivery efficiency, activation kinetics, imaging contrast, and tumor applicability, providing a novel approach for constructing high-contrast, high-specificity tumor imaging platforms.

The bacteria-based imaging approach effectively overcomes intertumoral heterogeneity by exploiting bacterial penetration into the tumor core rather than solely targeting surface antigens, thereby achieving broader and more consistent imaging outcomes. The inherent tropism of EcN for hypoxic tumor regions enables its efficient colonization across multiple cancer types, highlighting its broad applicability for pan-cancer imaging. As demonstrated above, a high S/N ratio of 9.94 was achieved by our bacteria-mediated extrinsic enzyme-activatable imaging system, compared to only 1.6 for the conventional dye Cy5, and signal stability was maintained for up to 2 h, fully covering the time requirements of routine fluorescence-guided surgery. The essential advantages of this system over non-activatable dyes were further revealed by *ex vivo* organ imaging. For Cy5, fluorescence signals in the lungs were observed to be comparable to those in the tumor, together with extremely strong accumulation in the kidneys (Fig. 7E and G). In sharp contrast, strong fluorescence was detected exclusively at the tumor site in the TMN-CPG/EcN–DSS–CPG2 system, and although detectable signals were also observed in the liver and kidneys, these were significantly lower than the tumor fluorescence, with negligible background in the lungs and other normal organs. This superior tumor retention and selectivity confer significant advantages to our strategy for fluorescence-guided surgery. These results further validate the feasibility of bacteria-mediated delivery, demonstrating that tumor imaging based on bacteria-delivered extrinsic enzyme-activatable fluorescent probes can be seamlessly integrated into existing clinical workflows, overcoming tumor heterogeneity and achieving high-contrast imaging with superior tumor selectivity.

The clinical translation of bacteria-based tumor imaging and therapy is gaining increasing momentum. For instance, the engineered EcN strain SYNB1891 (Synlogic) has completed a phase I clinical trial for the treatment of advanced solid tumors (NCT04167137), in which the safety of intratumoral injection of SYNB1891 as a monotherapy and in combination with atezolizumab was evaluated.^43^ In a complementary study, an engineered EcN strain carrying a synthetic arginine-nitric oxide circuit (EcN-NO) was constructed.^44^ This strain was shown to enable sustained intratumoral production of nitric oxide, thereby promoting vascular normalization and immune reprogramming, and enhancing the efficacy of anti-PD-L1 immunotherapy in multiple solid tumor mouse models. Additionally, EcN-based delivery systems have been developed for oral enzyme replacement therapy to reduce tumor growth.^45^ In the field of tumor imaging, fluorescently labeled EcN has been shown to selectively home to multiple tumor types without significant accumulation in healthy organs.^46^ These findings collectively support the clinical viability of the EcN–DSS–CPG2 system and emphasize the need for continued development to facilitate its transition into clinical oncology.

## Conclusions

In summary, an extrinsic enzyme-activatable fluorescent probe was developed to enable high-specificity, real-time imaging of malignant tumors. By leveraging the directional colonization of engineered probiotics, an EcN–DSS–CPG2 complex was constructed to enrich CPG2 specifically within tumors. No denaturation was observed and native enzymatic function was retained after CPG2 conjugation. A near-infrared fluorescent probe, TMN-CPG, specifically activated by CPG2, was designed and synthesized. Upon enzymatic cleavage of the amide bond, TMN-CPG releases a strong fluorescence signal, enabling high-contrast and high-resolution imaging in complex physiological environments. Experimental results indicated that TMN-CPG exhibits high sensitivity and excellent selectivity for CPG2, along with good biocompatibility and photostability. Confocal imaging of 4T1 cells co-incubated with TMN-CPG and CPG2 produced significant fluorescence signals, demonstrating excellent imaging capability at the cellular level. Notably, in the 4T1 tumor-bearing mouse model, this system activated fluorescence within 30 min, enabling high-intensity tumor-specific imaging that meets the stringent requirements for real-time intraoperative pathological diagnosis in terms of speed and spatial resolution. This work provided an efficient and reliable new imaging tool for intraoperative *in situ* tumor imaging and laid a theoretical and technical foundation for developing bacteria-assisted external enzyme delivery systems. Future research may explore combination strategies using multiple extrinsic enzymes, clinically translatable bacterial vectors and integration with multimodal imaging to advance this technology toward clinical application.

## Ethical statement

All animal procedures were performed in accordance with the Guidelines for Care and Use of Laboratory Animals of Suzhou Institute of Biomedical Engineering and Technology, Chinese Academy of Sciences (SIBET, CAS) and were approved by the Animal Ethics Committee of SIBET, CAS.

## Author contributions

The manuscript was written through contributions of all authors. All authors have given approval to the final version of the manuscript.

## Conflicts of interest

There are no conflicts to declare.

## Supplementary Material

SC-017-D6SC00484A-s001

## Data Availability

The data that support the findings of this study are available in the supplementary information (SI) of this article. Supplementary information: detailed experimental methods and the NMR spectra of the compounds. See DOI: https://doi.org/10.1039/d6sc00484a.
