# Supplementary material for: An extrinsic enzyme-activatable fluorescent probe for high-contrast tumor imaging
Source: Chem Sci. 2026 Apr 30;17(23):11672–81. doi: 10.1039/d6sc00484a (PMC13148267; doi:10.1039/d6sc00484a)
Supplement: SC-017-D6SC00484A-s001 [file SC-017-D6SC00484A-s001.pdf]

Supplementary Information (SI) for Chemical Science.
This journal is © The Royal Society of Chemistry 2026

Supplementary Material

An Extrinsic Enzyme-Activatable Fluorescent Probe for High-Contrast Tumor Imaging

Yifang Guo,[1,2] *Yuwei Du,*[1,2] *Liru Heng,*[2] *Huimin Miao,*[1,2] *Chen Chen,*[2] *Qiang Zhang,*[2] *Jiayi Wang,*[4] *Yige Shan,*[4] *Zhang Chen,**[2,3] *Li Li,**[1,2]

1. School of Biomedical Engineering (Suzhou), Division of Life Sciences and Medicine, University of Science and Technology of China, Hefei 230026, China.

2. Department of Biomaterials and Stem Cells, Suzhou Institute of Biomedical Engineering and Technology, Chinese Academy of Science (CAS), Suzhou 215163, China.

3. Jinan Guoke Medical Technology Development Co., Ltd., Jinan 250102, China.

4. School of Pharmacy, Xuzhou Medical University, Xuzhou 221004, China

Contents

1. Experimental Methods

2. Supporting Figures

1. Experimental Methods

Materials and instruments

All solvents and chemicals, unless special stated, were purchased commercially in analytical grade and used without further purification. All the reactions were performed under normal atmosphere and monitored by thin layer chromatography (TLC; Merck pre-coated silica gel 60 F254 plates). Chromatographic purification was performed on a silica gel (Macherey-Nagel, 230–400 mesh) column with the indicated eluent. ^{1}H and ^{13}C NMR spectra were recorded on a Bruker Avance NEO 600 MHz spectrometer at Kexuezhinanzhen institution (Hangzhou, China). Fluorescence spectra were collected using a Hitachi UV 2450 spectrophotometer (Tokyo, Japan). Confocal fluorescence images were performed on confocal laser scanning microscope (Nikon confocal microscope). *In vivo* fluorescence images were measured with Aivis-L3, Guoke Zhiying and IVIS Lumina III.

Synthesis of compound 2

Compound **1** (3 ml, 20 mM) and malononitrile (1.5 ml, 24 mM) were added to a solution of NH_4OAc (616.6 mg, 8 mmol) in 10 mL DMF. This mixture was stirred at 80 °C for 12 h and cooled down to room temperature. Then the mixture was extracted with ethyl acetate (50 mL). The organic layer was washed with saturated brine (30 mL), dried over anhydrous Na_2SO_4, filtered, and then evaporated under reduced pressure. The residue was purified by chromatography on a silica gel column using hexane/ethyl acetate (30:1 v/v) as the eluent to give compound **2** (3.54 g, 95%) as a yellow solid.

Synthesis of compound 4

A mixture of compound **2** (3.353 g, 18 mmol), compound **3** (3.231 g, 19.8 mmol) and 4-Methylpiperidine (223 μl, 1.8 mmol) in acetonitrile (40 mL) was stirred at 85 °C for 3 h under

reflux. The mixture was cooled to room temperature and then washed with acetonitrile (10 mL). The supernatant was removed by centrifugation, and the solid precipitate was vacuum-dried to obtain a light red solid (3.17 g, 53%), which was reserved for the next reaction without purification.

Synthesis of TMN-NH$_2$

Compound **4** (331.42 mg, 1 mM) was dissolved in 18 mL the mix solution of MeOH/HCl (v/v 1:2). This mixture was stirred at 105 °C under reflux overnight. After neutralization with 5% $NaHCO_3$, the solution was extracted by ethyl acetate, dried with anhydrous Na_2SO_4, and filtered. After removal of the solvent under reduced pressure by evaporation, the crude product was purified by silica column chromatography using hexane/ethyl acetate (3:1 v/v) as the eluent to afford the desired product TMN-NH$_2$ (154.3 mg, 53.3%) as a red solid.

^{1}H NMR (600 MHz, DMSO-d_6) δ 7.41 (d, J = 8.3 Hz, 2H), 7.18 (d, J = 15.9 Hz, 1H), 7.04 (d, J = 15.9 Hz, 1H), 6.71 (s, 1H), 6.57 (d, J = 8.2 Hz, 2H), 5.83 (s, 2H), 2.57 (s, 2H), 2.51 (s, 2H), 1.00 (s, 6H). Data were in agreement with those reported previously.[1]

Synthesis of compound 7

A solution of TMN-NH$_2$ (194.8 mg, 0.673 mM), compound **5** (162.9 mg, 0.808 mM) and compound **6** (298.6 mg, 1.01 mM) in a solution of DIPEA (353 μL) in 5 mL DCM was stirred for 2 h under room temperature. After washing $NaHCO_3$ and saturated brine, the solution was extracted by ethyl acetate. The organic layer was dried under reduced pressure by evaporation, the crude product compound **7** was obtained without further purification to the next step.

Synthesis of TMN-CPG

Compound **7** prepared above was dissolved in 6.73 mL the mix solution of TFA/H_2O (v/v 4:1) and stirred at room temperature for 3 h. After neutralization with NaOH, the solution was

washed with H_2O and extracted by ethyl acetate. The organic layer was dried under reduced pressure by evaporation, the product TMN-CPG was obtained which was redissolved in DMSO. This solution was purified by HPLC followed by lyophilization to give desired product TMN-CPG (58.9 mg, 18.9%) as a red-brown solid.

^{1}H NMR (600 MHz, DMSO-d_6) δ 9.30 (s, 1H), 7.57 (d, J = 8.9 Hz, 2H), 7.44 (d, J = 8.7 Hz, 2H), 7.23 (d, J = 2.7 Hz, 2H), 6.81 (s, 1H), 6.65 (d, J = 7.2 Hz, 1H), 4.01 (d, J = 6.7 Hz, 1H), 2.59 (s, 2H), 2.53 (s, 2H), 2.29 (d, J = 8.6 Hz, 1H), 2.21 (dd, J = 7.3, 2.3 Hz, 1H), 1.95 – 1.91 (m, 1H), 1.75 (dd, J = 8.1, 5.3 Hz, 1H), 1.01 (s, 6H). **^{13}C NMR** (151 MHz, DMSO-d_6) δ 174.5, 173.8, 170.3, 156.7, 154.3, 142.6, 138.2, 129.0, 128.5, 126.8, 121.6, 117.4, 114.2, 113.4, 74.9, 52.8, 42.4, 40.4, 38.2, 31.7, 28.9, 27.5 **HRMS** (ESI) m/z calcd for $C_{25}H_{27}O_5N_4^+$ $[M+H]^+$ 463.1976, found 463.1965.

Expression and Purification of CPG2

CPG2 was expressed and purified following the methods established previously.[2]

Lipophilicity Determination

The lipophilicity of the compounds was evaluated by partitioning them between 1 mL of octanol and 1 mL PBS. After 5 minutes of shaking and centrifugation at 5000 rpm for 10 minutes, Measuring the fluorescence intensity of the samples in each phase, and LogP was calculated as LogP = Log10[(y counts in octanol)/(y counts in PBS)]

Cell Culture and Cytotoxicity Assay

4T1 mammary gland carcinoma cells (4T1 cells) were purchased from Servicebio Technology Co., Ltd (Wuhan, China). 4T1 cells were propagated in petri dish cultured at 37 °C under a humidified 5% CO_2 atmosphere in DMEM medium, which were supplemented with 10% fetal bovine serum and 1% penicillin-streptomycin.

The cytotoxicity assay was performed using the water-soluble tetrazolium salt-1 (WST-1). Cells were seeded in a 96-well cell culture plate at a density of 1×10^4 cells per well and incubated for 24 h. Subsequently, the cells were treated with medium containing 0, 2, 4, 6, 8 and 10 μM of TMN-NH_2/TMN-CPG for 24 h, using cells without TMN-NH_2/TMN-CPG as control. Three replicate wells were used for each control and test concentration. Then, 10 μL of WST-1 reagent was added to each well, and the plate was incubated at 37 °C for 1 h under 5% CO_2 humidified atmosphere. Finally, the absorbance of the solution was measured at 450 nm using a microplate reader. Cell viability was expressed as a percentage of the control culture value, and it was calculated using the following equation:

$$\text{Cells viability (\%)} = (OD_{dye} - OD_{blank}) / (OD_{control} - OD_{blank}) \times 100$$

Confocal Laser Scanning Microscopy Imaging of Cancer Cells

Approximately 2×10^5 4T1 cells in DMEM (2 mL) were seeded on a glass-bottom confocal dish and allowed to adhere for 48 h at 37 °C in a humidified 5% CO_2 atmosphere. For the study of the extrinsic enzyme-activated fluorescence imaging system, the cells, after being rinsed with PBS, were incubated with different concentrations of enzymes in medium with 10 μM of Probe TMN-CPG for 30 min at the same condition. After removing the medium, the cells were rinsed with PBS twice and then treated with 1 mL of DMEM. Cells imaging was captured by using a confocal laser scanning microscope (Nikon confocal microscope) with a 60× oil immersion objective lens. The fluorescence signals of cells incubated with probes were collected at 663-738 nm channel upon excitation with a 488 nm laser. As a control, cancer cells were treated with 10 μM probe TMN-CPG and different concentrations of carboxypeptidase inhibitor (T10678) for 30 min at the same time, then imaged using confocal laser scanning microscopy.

***In vitro* Hemolysis Assay**

Hemolytic activity was evaluated using fresh blood. Red blood cells (RBCs) were isolated by centrifugation, washed three times with sterile PBS and reconstituted as a 2% (v/v) suspension. The RBC sus-pension was mixed with an equal volume of PBS (negative control), serially diluted bacterial cultures, or 1% Triton X-100 (positive control). After incubation at 37 °C for 1 h, samples were centrifuged and the absorbance of the supernatant was measured at 540 nm. Hemolysis percentage was calculated as: [(OD-sample - OD-PBS) / (OD-Triton X-100 - OD-PBS)] × 100%.

Construction of ECN-DSS-CPG2

EcN was purchased from Bio Sci Co., Ltd (Hangzhou, China). EcN cells were harvested by centrifugation (4,000 g, 10 min, 4 °C), washed three times with PBS, and resuspended in PBS to an OD_{600} of 1.0 for subsequent use on ice. A 1 mL aliquot of this suspension was then cross-linked with DSS at a final concentration of 1 mM and reacted on ice for 30 min with oscillation. The resulting mixture was centrifuged (4,000 g, 10 min) to remove unreacted DSS, washed twice with PBS, and resuspended in 1 mL PBS. CPG2 was then conjugated to the EcN activated by DSS by adding the enzyme at a final concentration of 5 μM and the mixture incubated at 4 °C for 2 h. Finally, the EcN-DSS-CPG2 conjugates were collected via centrifugation (4,000 g, 10 min), washed three times with PBS to remove unbound enzyme, and resuspended in 1 mL PBS.

***In vivo* imaging**

Female Balb/c nude mice (6-8 weeks old) were maintained in a pathogen-free environment under a 12/12 h light-dark cycle. All animal experiments were approved by the Animal Experimentation Ethics Committee of Chinese Academy of Sciences (SIBET, CAS [2025-C008]). The mice were kept under a pathogen-free condition with free access to food and water.

To establish mouse tumor models, Balb/c mice were inoculated with 4T1 cells (2.5×10^6 cells per mouse) under the armpit. A stock solution of TMN-CPG (2 mM) was prepared by dissolving it in PBS. After 14 days inoculation, 150 µL of the TMN-CPG prepared before or PBS was injected through the tail vein. After 30 min, the EcN-DSS-CPG2 solution (125 µL) was injected intratumorally. The fluorescence image of the mice was captured after the injection at different time points using two small animal in vivo imaging systems (Aivis-L3, Guoke Zhiying and IVIS Lumina III). Three mice were used for each group.

Histopathological Examination

4T1 tumor-bearing mice were euthanized 24 hours after probe injection. Normal organs and tumor tissues were excised, fixed in 4% paraformaldehyde, and subjected to H&E staining for histopathological examination.

Statistical Analysis

To ensure experimental accuracy, a minimum of three replicates were performed. The data are reported as the mean ± standard deviation and ample size (n) for each statistical analysis was represented in the corresponding figure legends. Statistical analyses were carried out using Origin 2018 and GraphPad Prism 8.0 software. The two-tailed Student's t-test was employed to assess the statistical significance between the two groups. p value of less than 0.05 was considered significant. $^*p < 0.05$, $^{**}p < 0.01$, and $^{***}p < 0.001$.

References

[1] Li, H.; Yao, Q.; Sun, W.; Shao, K.; Lu, Y.; Chung, J.; Kim, D.; Fan, J.; Long, S.; Du, J.; Li, Y.; Wang, J.; Yoon, J.; Peng, X. Aminopeptidase N Activatable Fluorescent Probe for Tracking Metastatic Cancer and Image-Guided Surgery via *in Situ* Spraying. *J. Am. Chem. Soc.* **2020**, *142* (13), 6381–6389.

[2] Chen, Z.; Yan, H.; Ma, H.; Gao, L.; Luo, G.; Li, L.; Lu, L.; Dong, W.; Zhang, L. Development of an Adenosine Deaminase-Resistant Cordycepin Prodrug Activated by Pseudomonas Carboxypeptidase G2. *Bioorganic Chem.* **2025**, *162*, 108596.

2. Supporting Figures

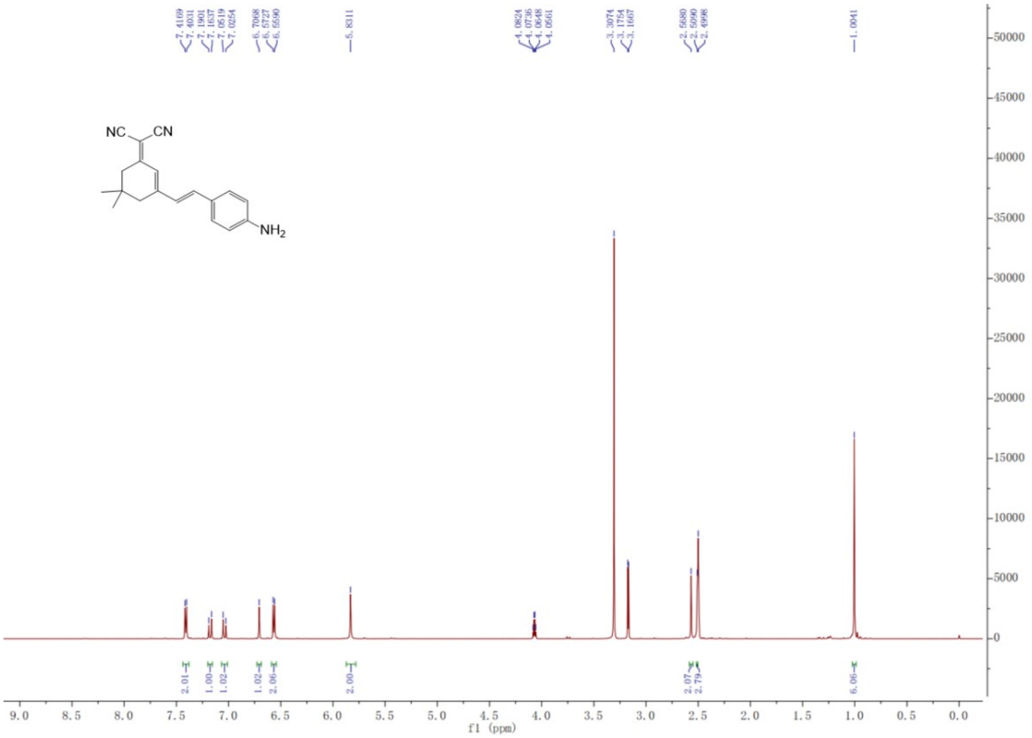

Figure S1. ^{1}H NMR spectrum (600 MHz) of TMN-NH_2 in DMSO-d_6.

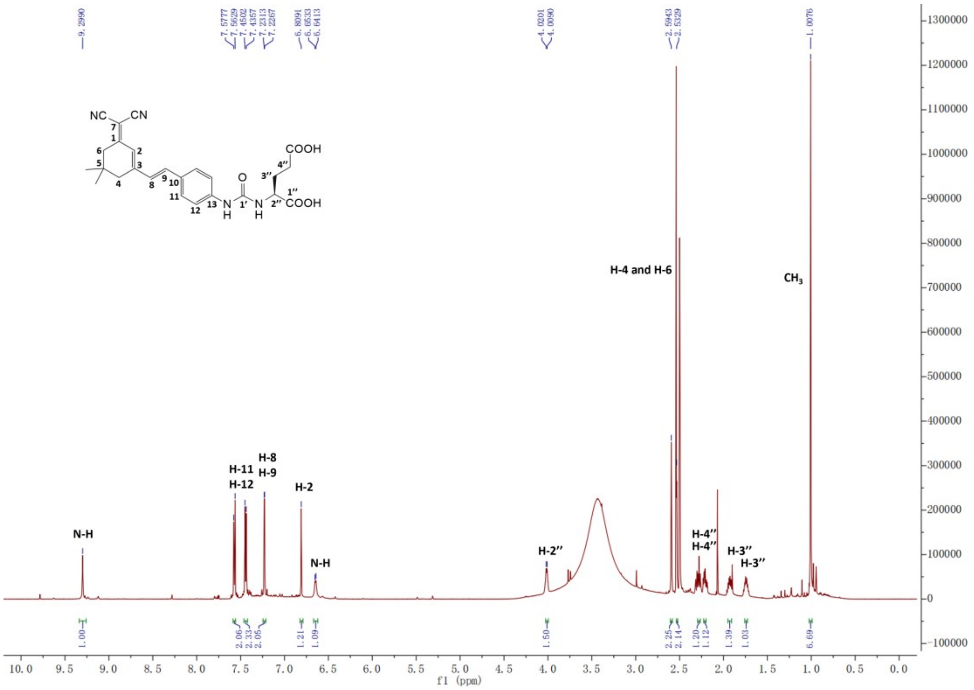

Figure S2. ^{1}H NMR spectrum (600 MHz) of TMN-CPG in DMSO-d_6.

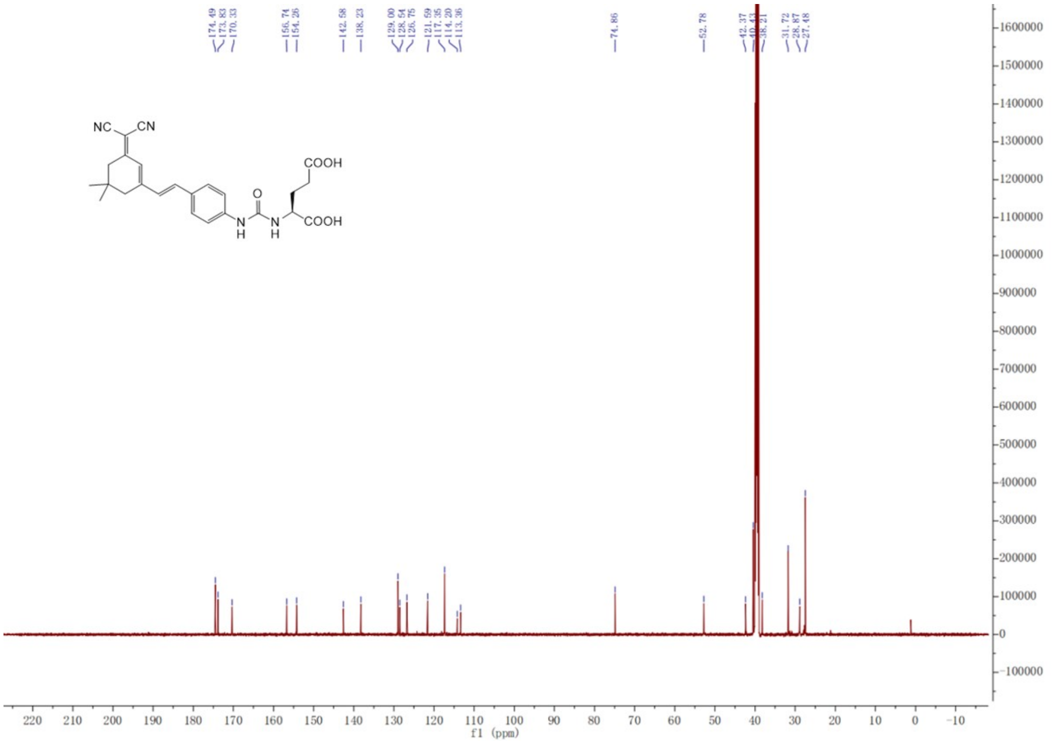

Figure S3. ^{13}C NMR spectrum (151 MHz) of TMN-CPG in DMSO-d_6.

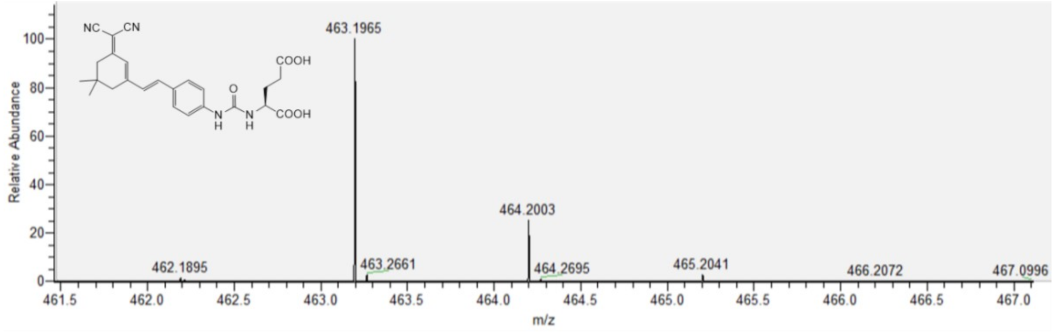

Figure S4. HRMS spectrum of TMN-CPG.

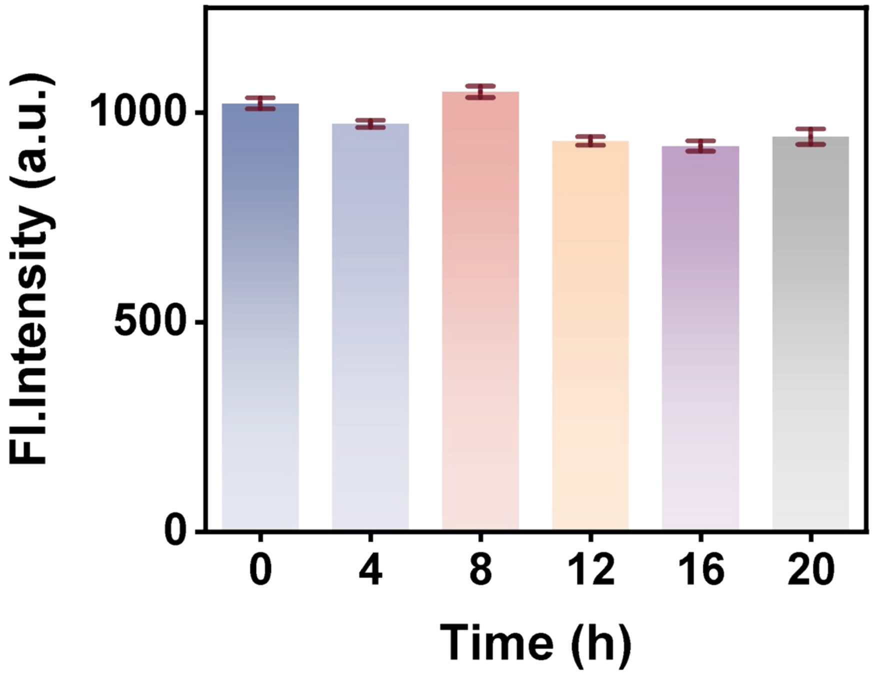

Figure S5. Fluorescence intensity of TMN-CPG (10 μM) were recorded in PBS solution as a function of time. Error bars are for $n = 3$.

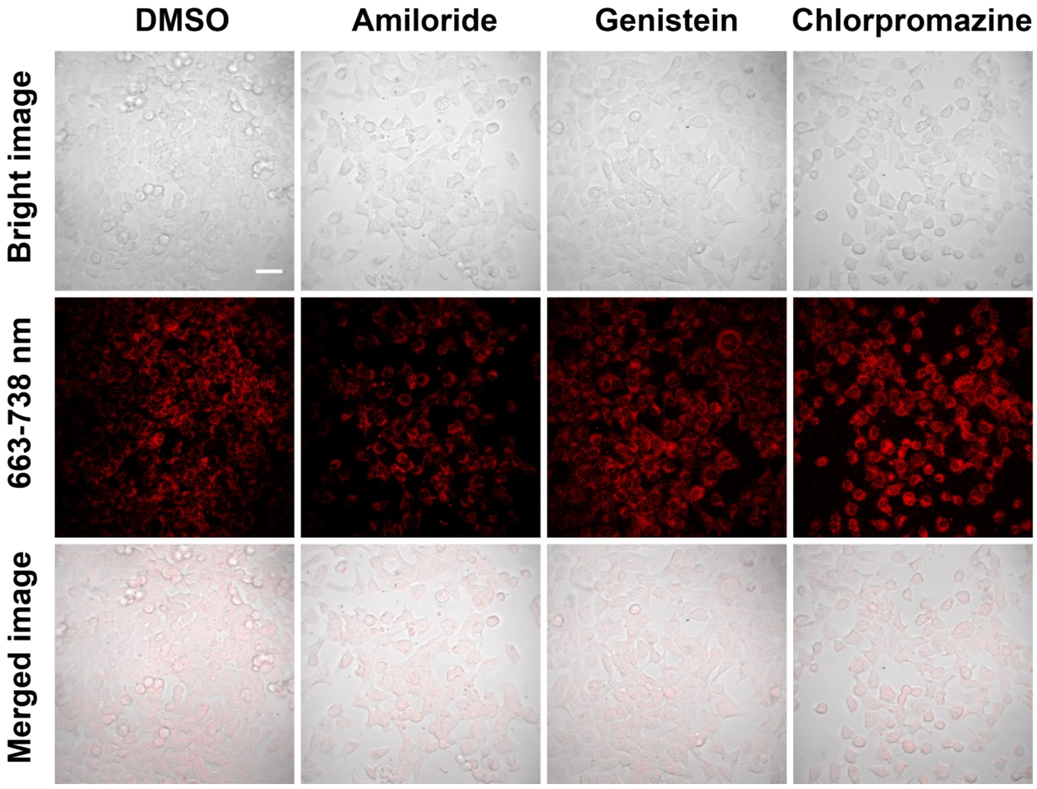

Figure S6. Confocal fluorescence images of 4T1 cells pre-treated with different inhibitors and incubated with TMN-NH_2 (10 μM) for 1 h. Scale bar = 50 μm.

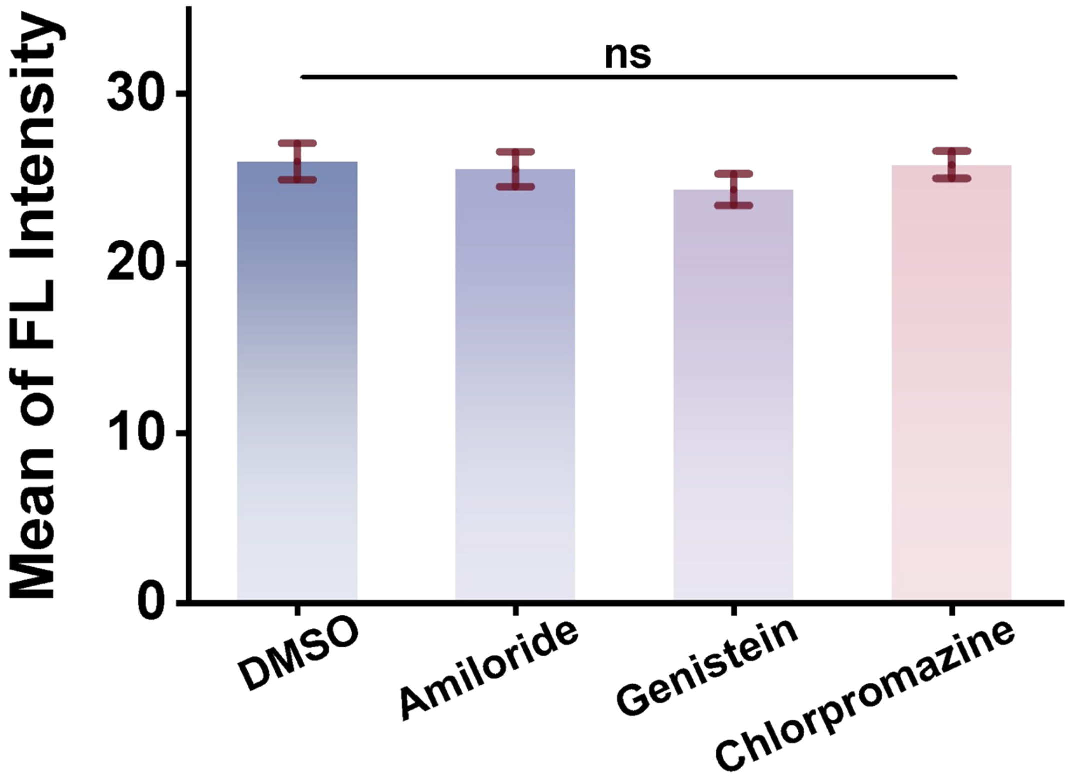

Figure S7. Histogram of the quantified FL intensities from 4T1 cells incubated with different inhibitors and incubated with TMN-NH_2 (10 μM) for 1 h. Error bars are for n = 3.

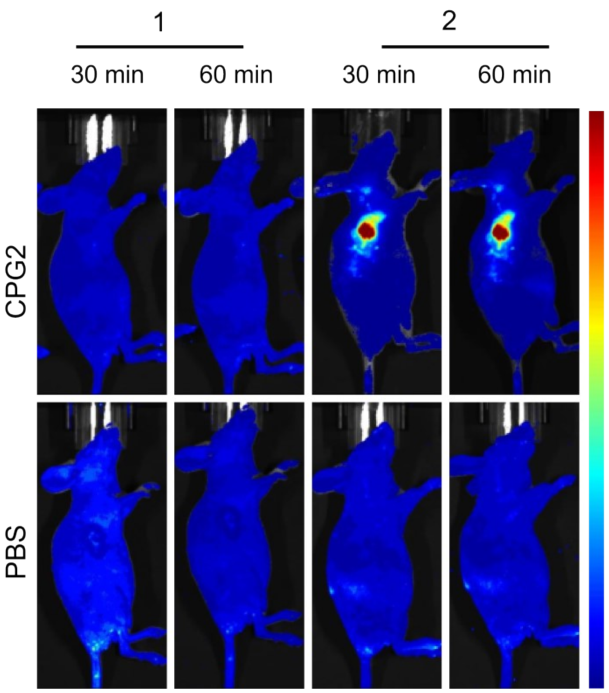

Figure S8. In vivo imaging of 4T1 xenograft-tumor-bearing nude Balb/c mice at 1 and 2 h after (1) intravenous (IV) injection of TMN-CPG and intratumoral (IT) injection of native CPG2 or PBS; (2) IV injection of native CPG2 or PBS and IT injection of TMN-CPG. λ_{ex} = 525 nm and $\lambda_{detection}$ = 670 nm.
